# Research Progress on the Pharmacological Effects and Network Regulatory Mechanisms of Pedunculoside

**DOI:** 10.3390/ijms27104475

**Published:** 2026-05-16

**Authors:** Xinxin Zhang, Huimin Li, Jiahui Li, Yijin Wu, Jingya Ruan, Qian Yao, Yi Zhang, Tao Wang

**Affiliations:** 1 School of Chinese Materia Medica, Tianjin University of Traditional Chinese Medicine, 10 Poyanghu Road, West Area, Tuanbo New Town, Jinghai District, Tianjin 301617, China; 18536554050@163.com (X.Z.); 15380711687@163.com (H.L.); 19153525979@163.com (J.L.); 15265767237@163.com (Y.W.); yq18812696707@163.com (Q.Y.); 2 Tianjin Key Laboratory of TCM Chemistry and Analysis, Tianjin University of Traditional Chinese Medicine, 10 Poyanghu Road, West Area, Tuanbo New Town, Jinghai District, Tianjin 301617, China; ruanjingya@tjutcm.edu.cn

**Keywords:** *Ilex chinensis* Sims, *Ilex rotunda* Thunb, pedunculoside, pharmacological effects, anti-inflammatory, network regulation, clinical translation

## Abstract

Pedunculoside (PE), a primary bioactive constituent of the traditional Chinese medicines (TCM) *Ilex chinensis* (*Sijiqing*) and *Ilex rotunda* (*Jiubiying*), has garnered growing interest for its broad pharmacological profile. This review synthesizes literature from 1999 to 2026, highlighting anti-inflammatory, cardioprotective, lipid-regulating, neuroprotective, and antitumor activities of PE. By modulating key signaling cascades, including NF-κB, MAPK, NLRP3, and Nrf2/HO-1, PE disrupts the interconnected “inflammation–oxidative stress–cell death” network, conferring multi-organ protection. These findings position PE as a key material basis underlying the *Qing Re Jie Du* properties of its parent herbs, while rationalizing their distinct clinical applications: *Sijiqing* for resolving swelling and blood stasis, and *Jiubiying* for promoting diuresis and alleviating pain. PE exhibits multi-target potential, directly or indirectly engaging proteins such as GATA6, TRAF6, and ACSL4, thereby contributing to formulation synergy. Despite its promise as a TCM-derived lead compound, PE research remains transitional, constrained by undefined direct molecular targets, limited pharmacokinetic data, and insufficient long-term safety profiling. Future efforts should integrate chemical biology and multi-omics to validate target engagement, systematically characterize pharmacokinetics and toxicology, and decode formulation synergy to accelerate clinical translation.

## 1. Introduction

Growing evidence indicates that anti-inflammatory activity underpins the broad therapeutic effects of many traditional Chinese medicines (TCM). Among these, the leaves of *Ilex chinensis* Sims (*Sijiqing*) and the bark of *Ilex rotunda* Thunb (*Jiubiying*), both from the Aquifoliaceae family, are clinically prominent. They are widely incorporated into compound formulas for respiratory and gastrointestinal disorders, as well as dermatological inflammation. *Sijiqing* is traditionally indicated for clearing heat, “detoxifying”, reducing swelling, and resolving blood stasis, whereas *Jiubiying* is traditionally used for heat clearance, “detoxification”, diuresis, and analgesia. Despite their distinct therapeutic focuses, both herbs are primarily used to treat swelling, pain, sores, and heat-toxin-induced diarrhea [1]. These traditional indications closely align with inflammatory pathologies recognized in modern medicine.

**Figure 1:**
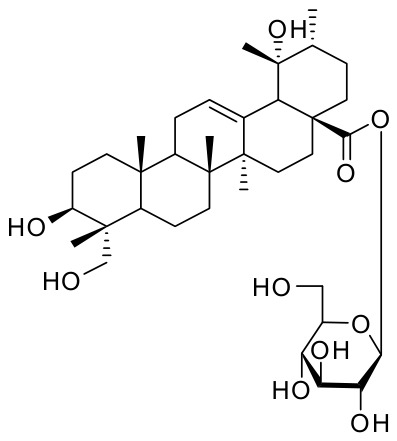
The structure of pedunculoside.

Phytochemical studies reveal that despite differences in botanical origin and medicinal parts, both herbs share an important triterpenoid saponin, pedunculoside (PE, Figure 1). PE serves as the chemical marker for *Jiubiying* and a key quality control indicator for *Sijiqing*. This compositional overlap strongly suggests that PE is the principal bioactive constituent responsible for their shared heat-clearing and “detoxifying” properties, which correspond to modern anti-inflammatory effects.

To date, pharmacological investigations of *Sijiqing* and *Jiubiying* have largely centered on crude extracts or compound preparations, demonstrating broad-spectrum activities such as anti-inflammatory [2,3,4,5,6,7], antibacterial [8,9,10], hepatoprotective [11,12], antiviral [13], antioxidant [14,15], antitumor [16,17], and cardioprotective [18] effects. In contrast, studies on isolated PE remain fragmented and lack systematic evaluation. Critical questions remain unresolved: Does PE constitute the molecular basis for the shared anti-inflammatory efficacy of these two herbs? Beyond inflammation, does PE also mediate their other documented protective effects? Addressing these gaps is essential for advancing the scientific understanding of these traditional remedies.

To address these questions, this review systematically compiles and analyzes literature on PE published between 1999 and 2026. We comprehensively summarize its pharmacological activities, with particular emphasis on anti-inflammatory, cardiovascular, lipid-lowering, neuroprotective, and antitumor effects, alongside their underlying molecular mechanisms. Furthermore, we evaluate the scientific rationale for positioning PE as the core active constituent of *Sijiqing* and *Jiubiying*. By elucidating the modern pharmacological basis of these classic heat-clearing and “detoxifying” herbs, this review aims to provide a theoretical foundation for future research, drug development, and clinical translation of PE-based therapeutics.

## 2. Materials and Methods

The review systematically collected references on the pharmacological effects of PE up to March 2026 from various electronic databases, including SciFinder, PubMed, and CNKI. The search combined the terms “pedunculoside”, “*Ilex chinensis*”, or “*Ilex rotunda*” with “pharmacological effects”, “pharmacological actions”, or “therapeutic effect”. Both Medical Subject Headings (MeSH) and keywords were used without language restrictions. To assess relevance, each author independently reviewed the titles and abstracts. After comparing the results, only the most relevant articles were included. Opinions, letters, and comments were excluded from the review.

## 3. Pharmacological Effects and Mechanisms of Pedunculoside

### 3.1. Anti-Inflammatory Effects

Inflammation is a physiological immune response triggered by infection, tissue injury, or harmful stimuli. While a controlled inflammatory response is essential for eliminating pathogens and facilitating tissue repair, excessive or dysregulated inflammation can lead to progressive tissue damage and organ dysfunction [19]. Consequently, the precise modulation of inflammatory processes has emerged as a critical therapeutic strategy. As the principal bioactive constituent of *Sijiqing* and *Jiubiying*, PE exhibits potent anti-inflammatory activity across diverse disease models. Its therapeutic efficacy is primarily mediated through the targeted regulation of multiple key inflammatory signaling pathways.

#### 3.1.1. Acute Lung Injury

Acute lung injury (ALI) is a diffuse inflammatory pulmonary disorder triggered by diverse pathogenic insults and characterized by disruption of the alveolar–capillary barrier [20]. Its pathogenesis involves complex interactions among pro-inflammatory cytokine release, NOD-like receptor family pyrin domain containing 3 (NLRP3) inflammasome activation, oxidative stress, and cellular apoptosis. Severe cases frequently progress to acute respiratory distress syndrome (ARDS), which can lead to respiratory failure and high mortality [21]. Accumulating evidence indicates that PE confers robust protection in experimental ALI models. Li et al. [22] demonstrated that oral administration of PE significantly ameliorated lung pathology in a mouse model of cecal ligation and puncture (CLP)-induced sepsis. PE treatment reduced inflammatory cell infiltration, alveolar septal thickening, and pulmonary congestion, while concurrently lowering the lung injury score, apoptotic cell rate, and wet-to-dry (W/D) lung weight ratio. Furthermore, PE markedly suppressed the release of interleukin (IL)-1β, IL-6, and tumor necrosis factor (TNF)-α in bronchoalveolar lavage fluid (BALF). Mechanistically, these protective effects were mediated through inhibition of the protein kinase B/nuclear factor κB (AKT/NF-κB) signaling cascade, as evidenced by the reversal of PE-induced protection following co-administration of the AKT activator SC79. Similar findings were reported by Yang et al. [23] in a lipopolysaccharide (LPS)-induced ALI/ARDS model. Intragastric treatment with PE preserved alveolar architecture, attenuated pulmonary edema (indicated by reduced W/D ratio and BALF protein levels), and downregulated IL-1β, IL-6, and TNF-α expression alongside the oxidative stress marker malondialdehyde (MDA). In vitro studies using MLE-12 lung epithelial cells confirmed that PE inhibits phosphorylation of NF-κB p65 and inhibitor of nuclear factor kappa B alpha (IκBα). The anti-inflammatory, antioxidant, and anti-apoptotic activities of PE were partially abolished by NF-κB activators, confirming that NF-κB pathway inhibition is central to its lung-protective mechanism.

#### 3.1.2. Myocarditis

Myocarditis denotes myocardial inflammation triggered by infectious, autoimmune, or toxic insults, often resulting in cardiomyocyte injury and impaired cardiac function [24,25]. Chronic low-grade inflammation also drives metabolic cardiomyopathies such as diabetic cardiomyopathy (DCM), primarily through NLRP3 inflammasome activation, subsequent myocardial fibrosis, and functional deterioration [26]. Consequently, targeted modulation of myocardial inflammation represents a vital therapeutic strategy. PE exhibits pronounced cardioprotective effects in both acute and chronic inflammatory cardiac models through distinct molecular mechanisms. Lu et al. [27] reported that PE significantly reduced IL-1β, IL-6, and TNF-α expression in both high-glucose-stimulated H9c2 cardiomyocytes and the myocardial tissue of DCM model rats following intragastric administration, while concurrently alleviating oxidative stress and apoptosis. This protection was mediated via activation of nuclear factor erythroid 2-related factor 2 (Nrf2) and its downstream targets, heme oxygenase-1 (HO-1) and NAD(P)H quinone dehydrogenase 1 (NQO1). The Nrf2 inhibitor ML385 abolished these benefits, confirming that PE exerts its anti-inflammatory and antioxidant effects through the Nrf2/HO-1 axis. In contrast, Zhuo et al. [28] identified a distinct mechanism in an LPS-induced acute myocarditis model. Tail vein injection of PE attenuated myocardial injury, improved cardiac function, and suppressed systemic and local TNF-α and IL-1β release. Molecular docking and functional assays revealed that PE directly binds to the purinergic P2X7 receptor (P2X7R), thereby blocking downstream NLRP3 inflammasome activation, phosphatidylinositol 4,5-bisphosphate (PIP2) signaling, and the mitogen-activated protein kinase (MAPK) cascade, including phosphorylation of p38, c-Jun N-terminal kinase (JNK), and extracellular signal-regulated kinase (ERK). These findings demonstrate that PE confers cardioprotection by targeting multiple critical inflammatory nodes.

#### 3.1.3. Colitis and Related Conditions

Ulcerative colitis (UC) is a chronic, relapsing inflammatory bowel disease driven by genetic susceptibility, environmental triggers, and gut microbiota dysbiosis. These factors compromise the mucosal barrier, dysregulate immune responses, and sustain chronic inflammation [29]. The persistent inflammatory milieu not only disrupts intestinal homeostasis but also substantially elevates the risk of colitis-associated colorectal cancer (CAC) [30]. Therefore, agents that modulate immune activation, restore barrier integrity, and resolve inflammation hold significant therapeutic value. As a key quality control marker for *Sijiqing* and *Jiubiying*, PE has attracted growing interest for its efficacy in UC and related pathologies.

*Ulcerative colitis.* Multiple studies confirm that both *Jiubiying* extracts and isolated PE ameliorate experimental UC. Li et al. [31] demonstrated that intragastric administration of *Jiubiying* water extract significantly attenuated dextran sulfate sodium (DSS)-induced UC in mice by restoring mucosal barrier function, reducing oxidative stress and inflammation, and modulating the oncostatin M/oncostatin M receptor (OSM/OSMR) axis. Given that PE is a major bioactive constituent of *Jiubiying*, it likely serves as a primary molecular mediator of these therapeutic effects. Further integrative pharmacological and transcriptomic investigations identified PE, ziyuglycoside II, and syringin as the core active components responsible for *Jiubiying*’s anti-UC activity [32]. Although PE monotherapy showed limited efficacy in reversing gross colonic pathology, it effectively reduced neutrophil infiltration and local inflammation. Notably, co-administration of PE and syringin synergistically enhanced the therapeutic impact of ziyuglycoside II. Transcriptomic profiling revealed that this combination exerts its effects by coordinately suppressing chemokine and cytokine overexpression within the cytokine–cytokine receptor interaction pathway. Liu et al. [33] directly evaluated gavage administration of PE in DSS-induced UC mice and observed dose-dependent reductions in the disease activity index (DAI), restoration of colon length, amelioration of histopathological damage, and decreased myeloperoxidase (MPO) activity. Mechanistically, PE suppressed LPS-induced macrophage activation and downregulated IL-1β, IL-6, and TNF-α production. These effects were mediated through inhibition of AKT, ERK1/2, JNK1/2, and p38 phosphorylation, thereby blocking the MAPK and AKT/NF-κB signaling cascades and curtailing inflammatory mediator synthesis. Additionally, PE has been identified as a core bioactive constituent of *Kuijieling* decoction, a TCM formula with demonstrated efficacy in UC management, as confirmed by LC-MS profiling [34,35]. This finding further supports the translational relevance of PE in UC therapy.

*Colitis-associated colorectal cancer* PE also exhibits potent anti-inflammatory and antineoplastic activity in CAC models. In an azoxymethane (AOM)/DSS-induced CAC mouse model, PE via intragastric gavage ameliorated colonic mucosal damage, reduced dysplastic lesions, and downregulated key inflammatory and oncogenic markers. In vitro experiments demonstrated that PE inhibits proliferation of HCT-116 human colorectal cancer cells and reverses the downregulation of ten–eleven translocation 3 (TET3) protein induced by miR-29a overexpression. Mechanistic analyses revealed that PE suppresses miR-29a expression, thereby upregulating its direct target, TET3, at both mRNA and protein levels. Concurrently, PE inhibits signal transducer and activator of transcription 3 (STAT3) activation and phosphorylation, effectively disrupting downstream inflammatory signaling. This dual action operates through regulation of the miR-29a/STAT3/TET3 axis [36], highlighting its potential to intervene in the inflammation-to-carcinoma transition via multi-target mechanisms.

#### 3.1.4. Other Inflammatory Diseases

*Mastitis* PE exhibits marked anti-inflammatory effects in experimental mastitis. Kan et al. [37] found that intragastric PE treatment inhibits AKT phosphorylation at ASP-184 site, thereby blocking downstream NF-κB and MAPK activation, which leads to significant reductions in TNF-α, IL-6, IL-1β, MPO, and inducible nitric oxide synthase (iNOS). Furthermore, PE upregulates the tight junction proteins occludin and claudin-3, facilitating restoration of the blood–milk barrier and mitigating mammary tissue inflammation.

*Rheumatoid arthritis* PE demonstrates multiple anti-inflammatory and joint-protective effects in rheumatoid arthritis models. Ma et al. [38] reported that intragastric administration of PE significantly suppresses the proliferation and migration of primary fibroblast-like synoviocytes (FLSs) and reduces the expression of IL-1β, IL-6, IL-8, and TNF-α. Mechanistically, PE attenuates TNF-α-induced activation of p38 and ERK. In a collagen-induced arthritis rat model, PE treatment significantly alleviates synovial inflammation and inhibits bone destruction, suggesting its potential therapeutic value for rheumatoid arthritis.

*Mouth ulcers* Topical PE application to the oral mucosa also has potential therapeutic effects in promoting the healing of oral ulcers in mice. The mechanism involves downregulating pro-inflammatory factors such as IL-6 and TNF-α, while upregulating the STAT3 and Mothers against decapentaplegic homolog 3 (Smad3) signaling pathways, thereby promoting collagen synthesis and accelerating the repair of the oral mucosa [39].

**Figure 2:**
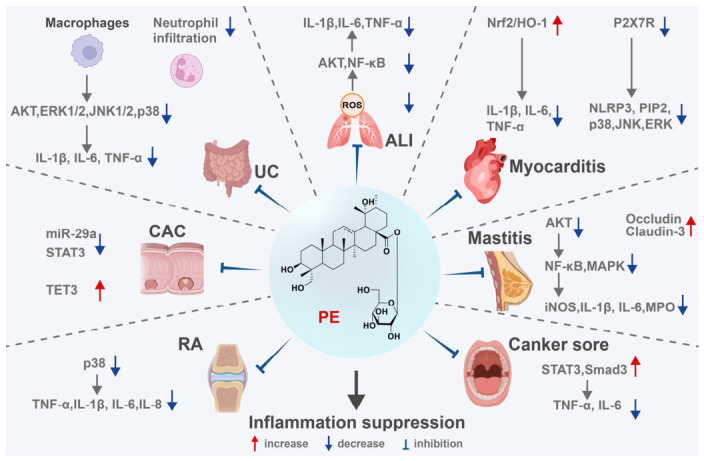
PE exhibits alleviating effects on inflammatory-related diseases. This figure was designed and edited using Adobe Illustrator, with basic schematic elements sourced from BioRender. Created in BioRender. Zhang, X. (2026) https://BioRender.com/q9n0gzr (accessed on 11 May 2026).

In summary, PE exhibits robust anti-inflammatory efficacy across diverse pathological models, including ALI, myocarditis, colitis, mastitis, rheumatoid arthritis, and oral ulcers. Its therapeutic actions are primarily mediated through modulation of key signaling cascades, notably the NF-κB, MAPK, Nrf2/HO-1, and P2X7R/NLRP3 pathways. By suppressing inflammatory mediator release, mitigating oxidative stress, and preserving tissue barrier integrity, PE effectively resolves inflammatory pathology (Figure 2). Nevertheless, the current body of evidence remains limited, and further studies are warranted to validate these preliminary findings and to explore additional mechanisms.

### 3.2. Cardiovascular Protective Effects

#### 3.2.1. Anti-Ischemic Effects

Myocardial ischemia is a multifactorial syndrome involving microvascular dysfunction, coronary spasm, and metabolic abnormalities, rather than being solely caused by obstructive coronary artery stenosis [40]. In clinical practice, reperfusion therapy aimed at restoring blood flow to ischemic myocardium often paradoxically exacerbates injury, a phenomenon known as myocardial ischemia/reperfusion injury (MIRI). MIRI is characterized by reactive oxygen species (ROS) bursts, calcium overload, inflammatory activation, and regulated cell death (e.g., ferroptosis, necroptosis, and pyroptosis) [41]. Therefore, effective treatment must address both the multifactorial nature of ischemia and the secondary damage incurred during reperfusion.

As a natural multi-target compound, PE has shown protective effects in both contexts. In an acute myocardial ischemia model, intragastric PE intervention significantly suppressed vasopressin-induced ECG J-point and T-wave abnormalities, reducing their amplitude and area under the curve, thereby demonstrating clear anti-myocardial ischemia activity [42]. In a coronary artery ligation-induced MIRI model, oral PE supplementation improved cardiac function parameters, increased left ventricular systolic pressure (LVSP) and the maximum rate of pressure rise in the left ventricle during isovolumic contraction (+dP/dtmax), while reducing left ventricular end-diastolic pressure (LVEDP) and the maximum rate of pressure decline (-dP/dtmax). In addition, PE decreased myocardial infarct size and cardiac index, elevated serum superoxide dismutase (SOD) activity, and reduced levels of MDA and cardiac troponin I (cTnI), indicating attenuation of oxidative stress and myocardial damage [43].

#### 3.2.2. Anti-Cardiotoxic Effects

Drug-induced cardiotoxicity (DICT) is a major limitation in the clinical use of several anticancer agents. The anthracycline doxorubicin (DOX) has attracted significant attention due to its dose-dependent cardiac toxicity, primarily driven by ROS bursts, mitochondrial dysfunction, and subsequent ferroptosis in cardiomyocytes [44]. In a DOX-induced cardiotoxicity model, intraperitoneal injection of PE significantly improved cardiac function, as evidenced by increased left ventricular ejection fraction (LVEF), left ventricular fractional shortening (LVFS), and stroke volume (SV), alongside marked attenuation of myocardial histopathological damage. At the molecular level, PE downregulated the mRNA expression of pro-inflammatory and cardiac stress markers, including prostaglandin-endoperoxide synthase 2 (*PTGS2*), *IL-1β*, and natriuretic peptide B (*Nppb*). Furthermore, PE reversed the DOX-induced upregulation of acyl-CoA synthetase long-chain family member 4 (ACSL4) and PTGS2 protein levels in myocardial tissue while restoring the expression of key iron-regulatory proteins, namely ferroportin 1 (FPN1), ferritin heavy chain 1 (FTH1), and ferritin. Collectively, these findings indicate that PE effectively mitigates lipid peroxidation and intracellular iron accumulation, thereby suppressing cardiomyocyte ferroptosis and attenuating inflammatory responses [45].

#### 3.2.3. Anti-Hypertrophic Effect

Pathological cardiac hypertrophy is an adaptive response to various cardiovascular stresses. Although initially compensatory, persistent stress can drive adverse cardiac remodeling and ultimately heart failure [46]. Intervening in this progression is therefore clinically important. Studies using transverse aortic constriction (TAC) and isoprenaline (ISO)-induced hypertrophy models have shown that intragastric PE intervention significantly alleviates myocardial hypertrophy, fibrosis, and cardiac dysfunction. Mechanistically, PE directly binds to and inhibits the nuclear translocation and transcriptional activity of the transcription factor GATA-binding protein 6 (GATA6), thereby downregulating the expression of fetal gene *Nppa* and *Nppb* [47].

**Figure 3:**
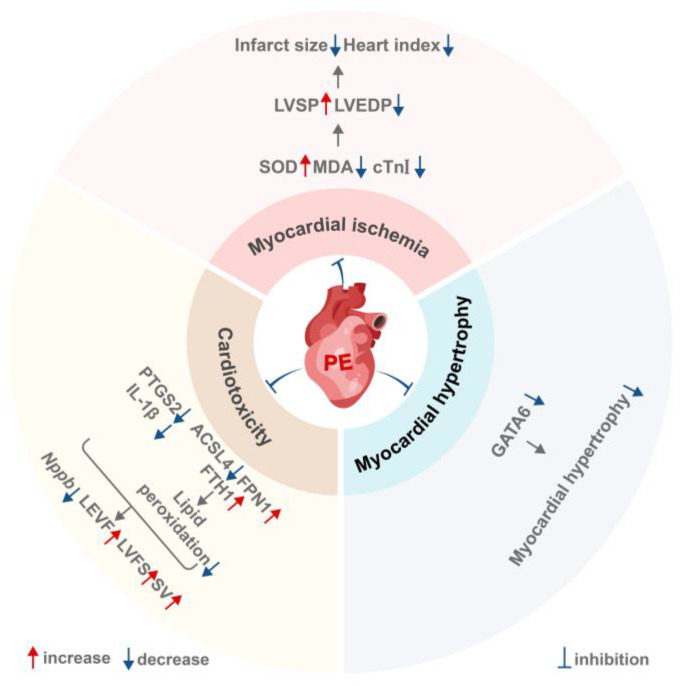
PE exhibits cardiovascular protective effects. This figure was edited in Adobe Illustrator.

In summary, PE has demonstrated significant beneficial effects in various cardiovascular disease models, including myocardial ischemia, cardiotoxicity, and cardiac hypertrophy. Its protective mechanisms exhibit multi-target characteristics: in myocardial ischemia, it primarily mitigates damage by combating oxidative stress; in cardiotoxicity, it inhibits ferroptosis by regulating key proteins such as ACSL4 and FTH1; in cardiac hypertrophy, it targets the transcription factor GATA6, inhibiting its nuclear translocation and transcriptional activity, thereby blocking the progression of pathological myocardial hypertrophy (Figure 3). These findings provide experimental evidence for the cardiovascular protective effects of PE. However, direct targets of PE require further confirmation, and the mechanisms underlying its interactions with proteins such as GATA6 and ACSL4 remain to be fully elucidated.

### 3.3. Lipid-Lowering Effect

Hyperlipidemia (HLP) is a pathological condition resulting from lipid metabolism disorders, characterized by persistent elevations in plasma lipids due to abnormalities in lipid transport and metabolism. Its main clinical features include elevated levels of serum total cholesterol (TC), triglycerides (TG), and low-density lipoprotein cholesterol (LDL-C), or reduced high-density lipoprotein cholesterol (HDL-C) [48]. Dyslipidemia is a major risk factor for metabolic diseases such as atherosclerosis, coronary heart disease, and fatty liver disease. Despite the availability of current lipid-lowering therapies, significant clinical challenges persist. Statins, the therapeutic cornerstone, are often limited by risks of myotoxicity and hepatotoxicity, leading to intolerance in certain patient populations. Fibrates are frequently associated with gastrointestinal disturbances and drug interactions, compromising long-term adherence. Consequently, there is an urgent need for novel therapeutic agents with improved efficacy and safety profiles to overcome these limitations. Developing safe and effective lipid-lowering drugs therefore remains a key research priority. As a natural bioactive compound, PE has demonstrated lipid-lowering activity in various animal and cellular models, acting through multi-target and multi-pathway mechanisms.

The 70% ethanol extract of *Jiubiying* administered by intragastric gavage significantly improves blood lipid levels in rats with HLP induced by a high-fat diet; given that the extract contains as much as 139.10 mg/g of PE, it is likely that PE is the primary active component responsible for the lipid-lowering effects of *Jiubiying* [49]. Early studies have shown that continuous intragastric administration of PE (40 mg/kg) for 14 days significantly reduces serum TC, LDL-C levels, and the atherosclerosis index in rats with diet-induced HLP, while simultaneously increasing HDL-C levels. In a Triton WR-1339-induced acute HLP model, PE (75 mg/kg) also lowered serum TC and total lipids. Mechanistic investigations suggested that these effects may involve inhibition of cholesterol synthesis [50].

Liu et al. [51] further elucidated the molecular mechanisms of PE in lipid metabolism. They found that PE promotes fatty acid β-oxidation by upregulating peroxisome proliferator-activated receptor alpha (PPAR-α), while simultaneously downregulating sterol regulatory element-binding protein 1 (SREBP-1), fatty acid synthase (FAS), and stearoyl-coenzyme A desaturase 1 (SCD-1) to inhibit lipid synthesis. Following intragastric gavage of PE for 7 weeks, this dual regulatory effect effectively reduced serum and hepatic TC and LDL-C levels in rats in a high-fat diet-induced model, and alleviated hepatic steatosis and epididymal fat accumulation. In 3T3-L1 adipocytes, PE also inhibits lipogenesis by activating the adenosine monophosphate-activated protein kinase (AMPK) pathway to suppress the expression of PPAR-γ, CCAAT/enhancer-binding protein α (C/EBPα), and SREBP-1.

In addition, Yan et al. [52] reported that PE inhibits pancreatic lipase in vitro, with a half-maximal inhibitory concentration (IC_50_) of 80.8 μg/mL, suggesting that it may exert anti-obesity and lipid-lowering effects by reducing the hydrolysis and absorption of dietary fats. This mechanism of action is similar to that of orlistat, a commonly used weight-loss drug in clinical practice. However, the clinically available lipase inhibitor orlistat often causes various gastrointestinal adverse reactions and interferes with the absorption of fat-soluble vitamins, which limits its long-term clinical application. Compared with current lipid-lowering drugs, PE may be developed as a novel candidate for lipid regulation, demonstrating promising research and application prospects.

Recent research shows that in primary mouse hepatocytes exposed to free fatty acids (FFA), treatment with PE (50, 100 μM) dose-dependently reduces lipid accumulation and lowers TC and TG levels. In mouse models of metabolic dysfunction-associated steatotic liver disease (MASLD) induced by high-fat–high-cholesterol diet (HFHC) or high-fat diet (HFD), oral administration of PE (15, 30 mg/kg) significantly reduces serum TC and TG, alleviates hepatic lipid deposition, and improves hepatocyte steatosis. Mechanistically, PE directly binds to heterogeneous nuclear ribonucleoprotein A1 (HNRNPA1), enhancing the stability of *PPARα* mRNA and upregulating PPARα expression. This in turn promotes the expression of key enzymes involved in fatty acid β-oxidation, including carnitine palmitoyltransferase 2 (CPT2), acyl-CoA dehydrogenase medium chain (ACADM), and acyl-CoA dehydrogenase long chain (ACADL), thereby enhancing mitochondrial fatty acid β-oxidation [53].

**Figure 4:**
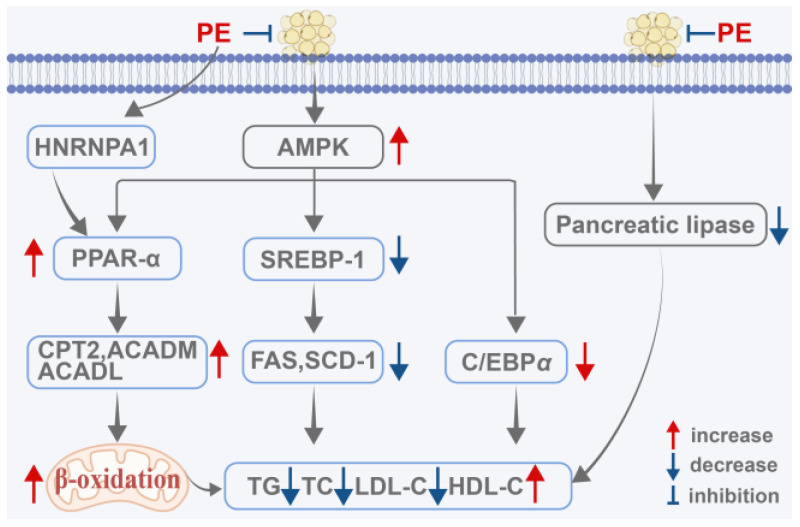
PE regulates lipid metabolism disorders. This figure was designed and edited using Adobe Illustrator, with basic schematic elements sourced from BioRen-der. Created in BioRender. Zhang, X. (2026) https://BioRender.com/q9n0gzr (accessed on 11 May 2026).

In summary, PE exhibits multi-target, multi-pathway regulation of blood lipid levels. Mechanistically, PE directly binds to HNRNPA1, enhancing *PPARα* mRNA stability and subsequently upregulating PPARα expression. This transcriptional activation drives the expression of key mitochondrial fatty acid β-oxidation enzymes (CPT2, ACADM, and ACADL), thereby accelerating lipid catabolism. Concurrently, PE suppresses endogenous lipogenesis by downregulating SREBP-1c, FAS, and SCD-1. It also attenuates adipocyte differentiation and prevents lipid accumulation via AMPK pathway activation. Furthermore, PE reduces intestinal lipid absorption through direct inhibition of pancreatic lipase, thereby limiting dietary fat uptake at the source (Figure 4). Collectively, these interconnected mechanisms establish a robust pharmacological foundation for PE’s efficacy in lipid metabolism disorders and underscore its therapeutic potential in the prevention and treatment of HLP and related metabolic diseases.

### 3.4. Neuroprotective Effects

#### 3.4.1. Regulatory Role of Neural Stem Cells

Neural stem cells (NSCs) possess self-renewal capacity and multipotent differentiation potential, playing a crucial role in repairing nervous system injuries and treating degenerative diseases. Modulating the proliferation and directed differentiation of endogenous NSCs has emerged as a key strategy in neural regenerative medicine. In vitro studies demonstrate that PE significantly promotes the proliferation of primary NSCs in a dose-dependent manner. Under differentiation-inducing conditions, PE enhances neuronal differentiation while simultaneously suppressing astrogenesis. Mechanistically, PE likely binds to phosphatidylinositol 3-kinase (PI3K), thereby activating the PI3K/AKT/glycogen synthase kinase-3β (GSK-3β) signaling pathway and promoting the phosphorylation of AKT and GSK-3β. This effect is abolished by the PI3K inhibitor LY294002, confirming strict pathway dependence. These findings indicate that PE can precisely modulate the fate of endogenous NSCs, offering a novel therapeutic strategy for neurodegenerative disorders [54].

#### 3.4.2. Neuroprotective Effects in Alzheimer’s Disease

Alzheimer’s disease (AD) is a progressive neurodegenerative disorder characterized by cognitive decline, β-amyloid (Aβ) deposition, neurofibrillary tangle formation, oxidative stress, and neuronal apoptosis. Targeting these interconnected pathological processes with multi-active compounds represents a promising therapeutic strategy. Recent evidence highlights the neuroprotective efficacy of PE in AD models. Li et al. [55] demonstrated that PE attenuates Aβ-induced apoptosis in PC12 cells, reduces intracellular ROS and restores mitochondrial membrane potential. In 3×Tg-AD transgenic mice, intraperitoneal injection of PE improved spatial learning and memory while attenuating hippocampal Aβ accumulation, neuroinflammation, and neuronal loss. Mechanistic investigations revealed that PE exerts these protective effects by activating the AMPK/GSK-3β/Nrf2 signaling pathway, thereby enhancing cellular antioxidant defenses and suppressing apoptosis. This work provides the first evidence that PE-mediated neuroprotection in AD is orchestrated through the AMPK pathway, supporting its potential as a multi-target therapeutic agent.

#### 3.4.3. Neuroprotective Effects in Neuropathic Pain

Neuropathic pain (NP) arises from sensory nervous system injury or disease and is characterized by exaggerated, stimulus-disproportionate pain responses. Central microglia play a pivotal role in NP pathogenesis [56]. In vitro, PE suppresses LPS-induced microglial activation by shifting the phenotype from pro-inflammatory M1 to anti-inflammatory M2. This phenotypic switch was characterized by a reduced proportion of CD32^+^ cells and decreased iNOS expression, concomitant with an increased proportion of CD206^+^ cells and upregulated arginase-1 (Arg-1), ultimately attenuating neuroinflammation. In a mouse model of chronic constriction injury (CCI), intraperitoneal PE injection could alleviate both mechanical and thermal hyperalgesia while similarly inhibiting spinal microglial M1 polarization and promoting M2 polarization. Mechanistic studies further revealed that these effects are mediated through the suppression of the Toll-like receptor 4 (TLR4)–NF-κB signaling pathway [57].

**Figure 5:**
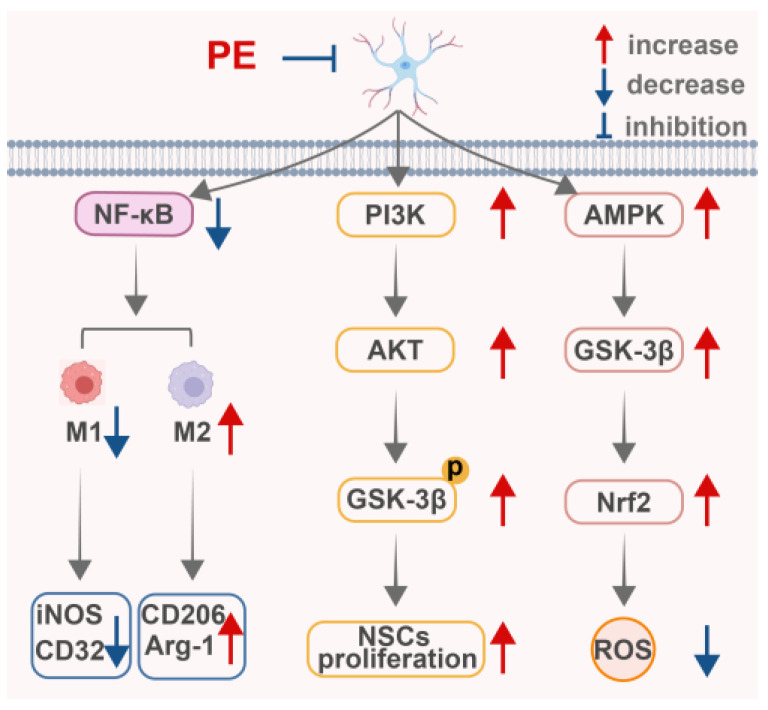
PE exhibits neuroprotective activities. This figure was designed and edited using Adobe Illustrator, with basic schematic elements sourced from BioRen-der. Created in BioRender. Zhang, X. (2026) https://BioRender.com/q9n0gzr (accessed on 11 May 2026).

In summary, PE exhibits multi-faceted neuroprotective properties (Figure 5). Beyond promoting endogenous NSC-mediated neural regeneration via PI3K/AKT/GSK-3β signaling, PE combats AD-related oxidative stress and apoptosis through AMPK/GSK-3β/Nrf2 activation, and attenuates neuropathic pain by modulating microglial polarization via TLR4/NF-κB inhibition. These complementary mechanisms highlight PE’s potential as a broad-spectrum agent for diverse central nervous system disorders. Nevertheless, its blood–brain barrier permeability, central pharmacokinetics, and long-term safety require comprehensive evaluation to facilitate clinical translation.

### 3.5. Antitumor Activity

Tumor progression is a complex, multistep process driven not only by intrinsic malignant proliferation but also by dynamic interactions within the tumor microenvironment (TME). Natural products have attracted considerable interest in oncology due to their multi-target mechanisms and favorable safety profiles. Accumulating evidence indicates that PE exerts broad antitumor effects through diverse mechanisms, including modulation of the tumor immune microenvironment, suppression of epithelial–mesenchymal transition (EMT), and reversal of chemoresistance.

#### 3.5.1. Bladder Cancer

Tumor-associated macrophages (TAMs) are a key component of the TME. M2-polarized TAMs secrete immunosuppressive cytokines that foster tumor growth, invasion, and metastasis, making macrophage repolarization a promising immunotherapeutic strategy. Using intraperitoneal injection of PE in mouse subcutaneous tumor models, Xu et al. [58] demonstrated that PE downregulates CD206 and inhibits M2 macrophage polarization. Moreover, PE significantly suppresses IL-4/IL-13-induced M2 polarization through inhibition of the tumor necrosis factor receptor-associated factor 6 (TRAF6)/NF-κB signaling axis. This intervention reduces the proportion of CD206 (MRC1)- and Arg-1-positive macrophages and decreases the secretion of immunosuppressive mediators such as transforming growth factor-β (TGF-β) and IL-10. In a bladder cancer (T24 and J82) and M2 macrophage co-culture system, PE treatment markedly attenuated cancer cell proliferation and invasiveness. Concurrently, PE restored epithelial characteristics by upregulating E-cadherin while downregulating the mesenchymal markers N-cadherin and vimentin. Functional validation confirmed that TRAF6 knockdown recapitulates the antitumor effects of PE, whereas TRAF6 overexpression abolishes them, underscoring the centrality of the TRAF6/NF-κB pathway in PE-mediated TME remodeling and indirect suppression of bladder cancer malignancy.

#### 3.5.2. Non-Small Cell Lung Cancer

EMT drives tumor invasiveness, metastasis, and acquired resistance to targeted therapies, positioning it as a critical therapeutic target. In in vitro assays using human non-small cell lung cancer (NSCLC) A549 cells, PE effectively reverses TGF-β1-induced EMT by upregulating E-cadherin and suppressing N-cadherin, vimentin, and the EMT transcription factors Slug and Snail. Notably, in gefitinib-resistant A549/GR cells, PE restores drug sensitivity and curbs migratory and invasive capacities. These effects are mediated through the ROS/MAPK/Nrf2 axis: PE lowers intracellular ROS levels, inhibits phosphorylation of ERK1/2, p38, and JNK, while suppressing the overexpression of Kelch-like ECH-associated protein 1 (Keap-1) and the activation of the Nrf2/HO-1 pathway. This coordinated modulation synergistically blocks EMT progression and re-sensitizes resistant cells to gefitinib [59].

#### 3.5.3. Hepatocellular Carcinoma

Hepatocellular carcinoma (HCC) ranks as the second leading cause of cancer-related mortality worldwide. Cyclin-dependent kinase 4 (CDK4) serves as a critical regulator of HCC progression and has emerged as a promising therapeutic target [60]. Zhou et al. [61] demonstrated that PE effectively inhibits HCC cell proliferation, induces apoptosis, and suppresses tumor growth and lung metastasis in vivo, without evident acute toxicity. Mechanistically, PE directly binds to and inhibits CDK4, resulting in G1/S phase cell cycle arrest. Concurrently, PE activates the p38 MAPK signaling pathway, upregulates pro-apoptotic Bcl-2-associated X protein (Bax), and downregulates anti-apoptotic B-cell lymphoma 2 (Bcl-2), thereby triggering mitochondrial-mediated apoptosis.

**Figure 6:**
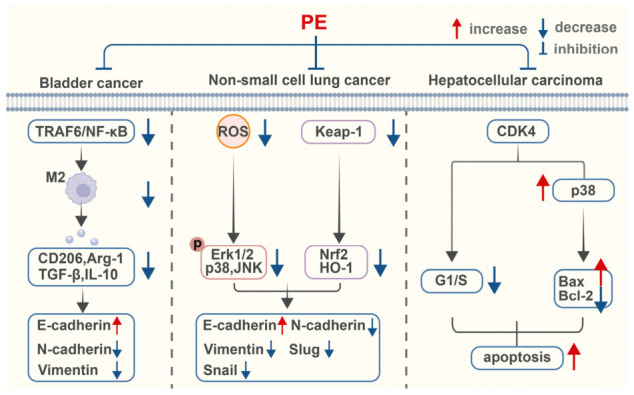
PE exhibits antitumor activities. This figure was designed and edited using Adobe Illustrator, with basic schematic elements sourced from BioRen-der. Created in BioRender. Zhang, X. (2026) https://BioRender.com/q9n0gzr (accessed on 11 May 2026).

Collectively, PE exhibits multi-target antitumor activity across distinct cancer types (Figure 6). In bladder cancer, PE inhibits M2 macrophage polarization by targeting the TRAF6/NF-κB signaling pathway, thereby reshaping TME and indirectly suppressing the malignant behavior of tumor cells. In NSCLC, PE reverses the EMT process by regulating the ROS/MAPK/Nrf2 signaling pathway, thereby restoring the sensitivity of drug-resistant cells to gefitinib. In HCC, PE directly targets and inhibits CDK4 to induce G1/S phase cell cycle arrest and activate the p38 MAPK pathway, ultimately inhibiting tumor progression. These findings expand the application prospects of PE in the field of cancer therapy and provide new insights for the development of cancer immunotherapy and drug resistance reversal strategies.

### 3.6. Other Pharmacological Effects

Beyond its previously described anti-inflammatory, cardioprotective, lipid-lowering, neuroprotective, and antitumor activities, PE has also exhibited hepatoprotective, myoprotective, and antiplatelet properties. Collectively, these findings expand the pharmacological profile of PE and provide a robust experimental foundation for its potential therapeutic applications across multiple clinical domains.

#### 3.6.1. Protective Effects Against Liver and Muscle Damage

Drug-induced hepatotoxicity and myotoxicity are prevalent adverse effects of clinical pharmacotherapy that significantly compromise patient quality of life and treatment adherence. The identification of compounds with dual hepatoprotective and myoprotective activities is therefore highly valuable for mitigating drug-related adverse events. Preclinical studies indicate that PE effectively ameliorates both chemical- or drug-induced liver injury and physical muscle damage. In hepatic injury models, PE significantly reduces serum aspartate aminotransferase (AST) and alanine aminotransferase (ALT) levels, attenuates liver fibrosis, and suppresses inflammatory cell infiltration. Similarly, in muscle injury models, oral gavage of PE prevents the elevation of serum creatine kinase (CK) and creatine kinase-MB (CK-MB) levels, while markedly improving myofiber disruption and interstitial edema. Notably, PE also demonstrates protective effects against simvastatin-induced concurrent hepatotoxicity and myotoxicity [62].

Given that liver fibrosis represents a pivotal stage in chronic liver disease pathogenesis and is driven largely by the aberrant activation of hepatic stellate cells (HSCs), further mechanistic investigations have elucidated the anti-fibrotic action of PE. Using integrated in vivo and in vitro approaches coupled with direct target validation, Wang et al. [63] demonstrated that PE directly binds to the transcription factor c-Jun. This interaction inhibits c-Jun phosphorylation and subsequent transcriptional activity, thereby disrupting pro-fibrotic signaling cascades, including the MAPK and NF-κB pathways. Consequently, PE effectively suppresses HSC activation and excessive collagen deposition, highlighting its potential as a targeted therapeutic agent for liver fibrosis.

#### 3.6.2. Antiplatelet Effect

Dysregulated platelet activation and aggregation underpin thrombus formation and represent a central pathological mechanism in cardiovascular and cerebrovascular disorders. Consequently, the development of safe and potent antiplatelet agents remains a critical therapeutic priority. Tan et al. [64] reported that in an in vitro rabbit platelet aggregation model, PE significantly inhibits adenosine diphosphate (ADP)-induced platelet aggregation, with an IC_50_ of 19.1 μM. Its activity was significantly stronger than that of the positive control drug ozagrel (IC_50_ = 65.2 μM). This finding suggests that PE may exert its antiplatelet aggregation effect by interfering with the ADP receptor signaling pathway. Furthermore, in a comprehensive phytochemical analysis of *Jiubiying*, Yang et al. [65] identified PE as a key triterpenoid constituent with documented antiplatelet activity, further corroborating its relevance among the antithrombotic phytochemicals derived from *Ilex* species.

**Figure 7:**
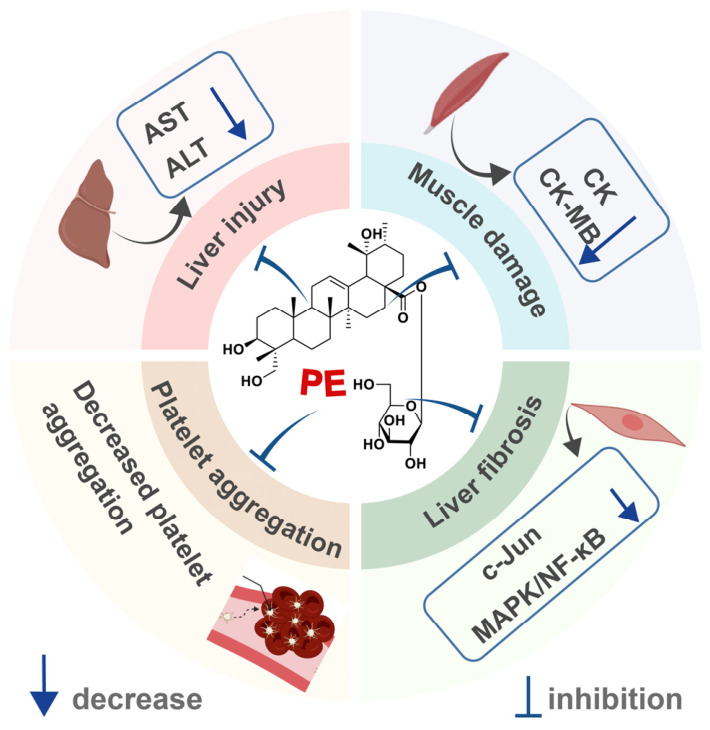
Beneficial effects of PE on other diseases. This figure was designed and edited using Adobe Illustrator, with basic schematic elements sourced from BioRen-der. Created in BioRender. Zhang, X. (2026) https://BioRender.com/q9n0gzr (accessed on 11 May 2026).

In summary, PE demonstrates significant hepatoprotective, myoprotective, and antiplatelet properties (Figure 7). It effectively lowers serum transaminases, mitigates hepatic fibrosis and inflammation, and directly targets the transcription factor c-Jun to suppress pro-fibrotic signaling. Concurrently, PE attenuates muscle damage by reducing circulating muscle enzymes and preserving myofiber integrity. Its antiplatelet potency, particularly against ADP-induced aggregation, notably exceeds that of ozagrel in vitro. Collectively, these findings broaden the therapeutic potential of PE and offer promising avenues for developing novel interventions against drug-induced organ toxicity, myopathies, and thromboembolic disorders.

## 4. Discussion

As a principal bioactive constituent of *Sijiqing* and *Jiubiying*, PE has garnered increasing attention owing to its broad-spectrum pharmacological activities. This review systematically summarizes current evidence on the anti-inflammatory, cardioprotective, hypolipidemic, neuroprotective, and antitumor effects of PE, highlighting its multi-target and multi-pathway mode of action. Despite the disease-specific manifestations of its therapeutic effects, these diverse activities converge on shared molecular mechanisms.

**Figure 8:**
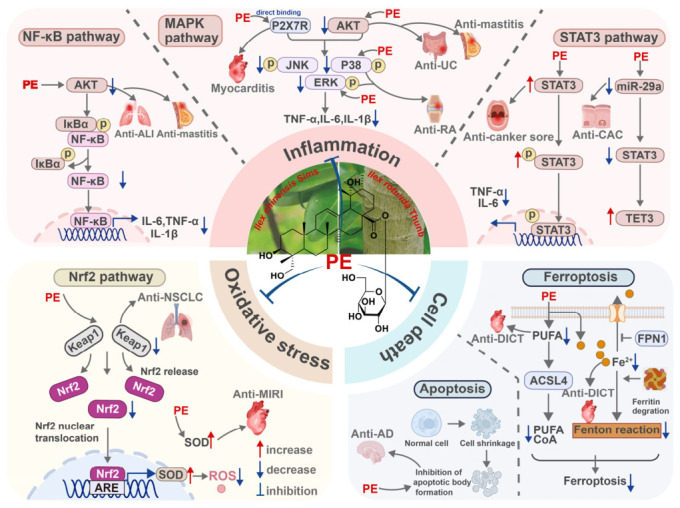
PE modulates the inflammatory–oxidative–death axis to achieve multi-organ protection. This figure was designed and edited using Adobe Illustrator, with basic schematic elements sourced from BioRen-der. Created in BioRender. Zhang, X. (2026) https://BioRender.com/q9n0gzr (accessed on 11 May 2026).

Mechanistic analyses reveal that PE exerts its systemic protective effects primarily by modulating the interconnected pathological triad of “inflammation–oxidative stress–cell death”. Inflammation serves as the central hub in this network. Across diverse disease models, including ALI, myocarditis, colitis, and mastitis, PE consistently suppresses canonical This figure was designed and edited using Adobe Illustrator, with basic schematic elements sourced from BioRender. Created in BioRender. Zhang, X. (2026) https://BioRender.com/q9n0gzr (accessed on 11 May 2026). Such as IL-1β, IL-6, and TNF-α. Given that these same pathways drive the pathogenesis of cardiovascular (e.g., MIRI) and neurological disorders (e.g., AD and neuropathic pain), the anti-inflammatory activity of PE likely constitutes a foundational mechanism underlying its broad therapeutic efficacy. Oxidative stress operates both as a downstream consequence of inflammation and as an independent pathological driver. PE mitigates oxidative damage by scavenging ROS, enhancing SOD activity, and reducing MDA accumulation. These effects are largely mediated through activation of the Nrf2/HO-1 pathway, which synergizes with anti-inflammatory signaling to disrupt the vicious cycle between oxidative stress and inflammation. Concurrently, PE exhibits context-dependent regulation of programmed cell death. It suppresses ferroptosis in cardiovascular models by modulating ACSL4 and FTH1 expression, inhibits apoptosis in neurodegenerative contexts, and alters tumor cell viability through microenvironmental remodeling. As illustrated in Figure 8, these three axes are highly crosstalk-dependent: inflammation acts as the primary trigger, while oxidative stress and cell death function as both downstream effectors and upstream amplifiers. By concurrently targeting this integrated network, PE achieves comprehensive multi-organ protection. However, it should be noted that these findings are based exclusively on preclinical studies, and clinical evidence supporting the multi-organ protective effects of PE in humans is currently lacking.

These pharmacological profiles position PE as a key material basis underlying the traditional “clearing heat and detoxifying” actions of both *Sijiqing* and *Jiubiying*. The broad-spectrum bioactivity of PE also offers a modern biological rationale for the distinct clinical indications of these two herbs: *Sijiqing* is traditionally used to “reduce swelling and invigorate blood”, whereas *Jiubiying* is indicated for “resolving dampness and alleviating pain”. From a contemporary perspective, the anti-inflammatory properties of PE directly correspond to the resolution of “heat-toxin,” as TCM manifestations such as swelling, pain, ulceration, and dysentery closely mirror acute inflammatory and infectious processes in modern pathophysiology. The demonstrated efficacy of PE in models of ALI, colitis, and myocarditis aligns with the TCM syndromes of lung-heat cough, heat-toxin dysentery, and heart-invading heat-toxin, respectively, thereby substantiating its role in mitigating “inflammatory toxicity”. Furthermore, PE mediates the differentiated therapeutic actions of the two herbs. In line with *Sijiqing*’s blood-invigorating properties, PE promotes vascular protection and microcirculatory improvement. Corresponding to *Jiubiying*’s dampness-resolving effects, PE modulates lipid metabolism and exerts hepatoprotective actions, while its anti-inflammatory and antidiarrheal activities have been empirically validated in colitis models. Thus, anchored by its core anti-inflammatory activity and complemented by cardiovascular and metabolic regulatory functions, PE serves as a multifunctional pharmacological carrier that bridges the shared and divergent therapeutic profiles of these two herbs.

A defining feature of PE is its multi-target engagement and potential for synergistic interactions within complex botanical matrices. PE interacts with diverse molecular targets across different pathological contexts, including GATA6 in cardiac hypertrophy, TRAF6 in bladder cancer, ACSL4/FTH1 in ferroptosis regulation, and PI3K in neural stem cell modulation. This pleiotropic targeting allows PE to simultaneously disrupt multiple nodes of disease progression, offering therapeutic advantages over conventional single-target agents. Moreover, the efficacy of PE in clinical practice is likely amplified through synergistic interactions within traditional formulas. For instance, in ulcerative colitis models, PE operates synergistically with ziyuglycoside II and syringin as part of the core bioactive constituent group of *Jiubiying*. It also serves as a principal active component in *Kuijieling* decoction. These observations underscore that the therapeutic impact of PE in polyherbal formulations depends on coordinated, multi-component network regulation rather than isolated action. Consequently, PE represents a scientifically justified candidate for use as a quality control marker in the standardization and pharmacopoeial evaluation of *Sijiqing*, *Jiubiying*, and related preparations.

Despite substantial progress in elucidating the pharmacological effects of PE, current research remains largely phenotype-driven, with several critical bottlenecks hindering its clinical translation: (1) Unverified direct targets. Mechanistic insights predominantly rely on indirect pathway modulation using pharmacological inhibitors or genetic manipulation. Direct binding interactions with putative targets, such as TRAF6 and ACSL4, remain unconfirmed. (2) Incomplete pharmacokinetic and toxicological profiling. The in vivo ADME properties of PE are poorly characterized. Key parameters, including oral bioavailability, metabolic fate, tissue distribution, and blood–brain barrier permeability, lack systematic investigation. Moreover, existing safety data are restricted to short-term efficacy models, with comprehensive assessments of chronic, reproductive, and genotoxicity still absent. (3) Formulation and translational barriers. The inherent limitations in solubility and stability of PE impede reliable bioavailability, while its therapeutic window and selective cytotoxicity between malignant and normal cells require rigorous quantification to establish a viable safety margin for clinical application.

## 5. Conclusions

In summary, PE, a principal bioactive constituent of the traditional Chinese medicines *Sijiqing* and *Jiubiying*, exhibits broad pharmacological activities, including anti-inflammatory, cardioprotective, hypolipidemic, neuroprotective, and antitumor effects. Mechanistically, PE modulates multiple signaling pathways (e.g., NF-κB, MAPK, PI3K/AKT, AMPK, and Nrf2) and disrupts the interconnected network of inflammation, oxidative stress, and cell death to confer multi-organ protection. These findings substantiate the therapeutic value of PE and support its application as a quality control marker for traditional formulations and as a promising lead compound for novel drug discovery.

To systematically address the aforementioned limitations and accelerate clinical translation, future research must prioritize three strategic directions. First, target deconvolution should be advanced through integrated chemical and structural biology approaches, employing techniques such as molecular docking, surface plasmon resonance, and cellular thermal shift assays to validate direct protein engagement and inform rational structural optimization. Second, comprehensive ADME and toxicological profiling is essential to establish definitive pharmacokinetic parameters, identify potential target organ toxicity, and determine the no observed adverse effect level. Third, formulation innovation and translational development should be accelerated by engineering advanced delivery systems, including liposomes, nanoparticles, and phospholipid complexes, to overcome solubility constraints and enhance systemic exposure, thereby facilitating preclinical advancement and early-phase clinical trials.

Through the integration of molecular pharmacology, chemical biology, medicinal chemistry, and pharmaceutics, PE research is transitioning from phenomenological observation to mechanistic validation and ultimately to clinical application. As a bioactive scaffold derived from traditional medicine, PE offers substantial potential to drive the development of next-generation therapeutics.

## Data Availability

No new data were created or analyzed in this study. Data sharing is not applicable to this article.

## Abbreviation

+dP/dtmax: maximum rate of pressure rise in the left ventricle during isovolumic contraction; -dP/dtmax: maximum rate of pressure decline in the left ventricle during isovolumic relaxation; ACADL: acyl-CoA dehydrogenase long chain; ACADM: acyl-CoA dehydrogenase medium chain; ACSL4: acyl-CoA synthetase long-chain family member 4; AD: Alzheimer’s disease; ADP: adenosine diphosphate; AKT: protein Kinase B; ALI: acute lung injury; ALT: alanine aminotransferase; AOM: azoxymethane; ARDS: acute respiratory distress syndrome; AST: aspartate aminotransferase; Arg-1: arginase-1; Aβ: β-amyloid; Bax: Bcl-2-associated X protein; Bcl-2: B-cell lymphoma 2; C/EBPα: CCAAT/enhancer-binding protein α; CAC: colitis-associated colorectal cancer; CCI: chronic constriction injury; CDK4: cyclin-dependent kinase 4; CK: creatine kinase; CK-MB: creatine kinase-MB; CLP: cecal ligation and puncture; CPT2: carnitine palmitoyltransferase 2; DAI: disease activity index; DCM: diabetic cardiomyopathy; DICT: drug-induced cardiotoxicity; DOX: doxorubicin; DSS: dextran sulfate sodium; EMT: epithelial–mesenchymal transition; ERK: extracellular signal-regulated kinase; FAS: fatty acid synthase; FFA: free fatty acids; FLSs: fibroblast-like synoviocytes; FPN1: ferroportin 1; FTH1: ferritin heavy chain 1; GATA6: GATA-binding protein 6; GSK-3β: glycogen synthase kinase-3β; HCC: hepatocellular carcinoma; HDL-C: high-density lipoprotein cholesterol; HFD: high-fat diet; HFHC: high-fat high-cholesterol; HLP: hyperlipidemia; HNRNPA1: heterogeneous nuclear ribonucleoprotein A1; HO-1: heme oxygenase-1; HSCs: hepatic stellate cells; IL: interleukin; ISO: isoprenaline; JNK: c-Jun N-terminal kinase; Keap-1: Kelch-like ECH-associated protein 1; LDL-C: low-density lipoprotein cholesterol; LPS: lipopolysaccharide; LVEDP: left ventricular end-diastolic pressure; LVEF: left ventricular ejection fraction; LVFS: left ventricular fractional shortening; LVSP: left ventricular systolic pressure; MAPK: mitogen-activated protein kinase; MASLD: metabolic dysfunction-associated steatotic liver disease; MDA: malondialdehyde; MIRI: myocardial ischemia/reperfusion injury; MPO: myeloperoxidase; NF-κB: nuclear factor κB; NLRP3: NOD-like receptor family pyrin domain containing 3; NP: neuropathic pain; Nppb: natriuretic peptide B; NQO1: NAD(P)H quinone dehydrogenase 1; NSCLC: non-small cell lung cancer; NSCs: neural stem cells; Nrf2: nuclear factor erythroid 2-related factor 2; OSM/OSMR: oncostatin M/oncostatin M receptor; P2X7R: P2X7 receptor; PE: pedunculoside; PI3K: phosphatidylinositol 3-kinase; PIP2: phosphatidylinositol 4,5-bisphosphate; PPAR-α: peroxisome proliferator-activated receptor alpha; PTGS2: prostaglandin-endoperoxide synthase 2; ROS: reactive oxygen species; SCD-1: stearoyl-coenzyme A desaturase 1; SOD: superoxide dismutase; SREBP-1: sterol regulatory element-binding protein 1; STAT3: signal transducer and activator of transcription 3; SV: stroke volume; Smad3: Mothers against decapentaplegic homolog 3; TAC: transverse aortic constriction; TAMs: tumor-associated macrophages; TC: total cholesterol; TCM: traditional Chinese medicines; TET3: ten–eleven translocation 3; TG: triglycerides; TGF-β: transforming growth factor-β; TLR4: Toll-like receptor 4; TME: tumor microenvironment; TNF: tumor necrosis factor; TRAF6: tumor necrosis factor receptor-associated factor 6; UC: ulcerative colitis; W/D: wet-to-dry; cTnI: cardiac troponin I; iNOS: inducible nitric oxide synthase.
